# Curcumol inhibits EMCV replication by activating CH25H and inhibiting the formation of ROs

**DOI:** 10.1186/s12917-022-03531-x

**Published:** 2022-12-26

**Authors:** Jiangang Zheng, Panpan Sun, Na Sun, Zhili Hao, Kuohai Fan, Wei Yin, Ajab Khan, Jianhua Guo, Xiaozhong Zheng, Hongquan Li

**Affiliations:** 1 Shanxi key lab for modernization of TCVM, College of Veterinary Medicine, Shanxi Agricultural University, Taigu, 030801 Shanxi P.R. China; 2 Laboratory Animal Center, Shanxi Agricultural University, Taigu, 030801 Shanxi China; 3 College of Veterinary Medicine, Jilin University, Changchun, Jilin, 130015 China; 4 Faculty of Veterinary and Animal sciences, the University of Agriculture, Dera Ismail Khan, Khyber Pakhtunkhwa 29050 Pakistan; 5 Department of Veterinary Pathobiology, Schubot Exotic Bird Health Center, Texas A&M University, College Station, TX 77843 USA; 6 Centre for Inflammation Research, Queen’s Medical Research Institute, The University of Edinburgh, EH164TJ, Edinburgh, UK

**Keywords:** EMCV, Curcumol, CH25H, Cholesterol, ROs

## Abstract

**Background:**

Zedoary turmeric oil extracted from the roots of curcuma (*Curcuma aeruginosa* Roxb.) is used for the treatment of myocarditis in China. EMCV infection causes abortion in pregnant sows and myocarditis in piglets. Our previous studies demonstrated that curcumol significantly increased the expression of IFN-β in EMCV infected HEK-293T cells. The present results showed that curcumol inhibits EMCV replication by interfering the host cell cholesterol homeostasis and reducing ROs production through activation of the JAK/STAT signaling pathway.

**Method:**

This study was designed to explore whether curcumol can inhibit the replication of encephalomyocarditis viruses (EMCV) in cell culture. The expression level of JAK1, IRF9, STAT2, P-STAT2, CH25H, PI4KA and OSBP in EMCV-infected HEK-293T cells treated with curcumol, ribavirin or hydroxypropyl-β-CD (HPCD) were determined by Western blotting (WB). The cholesterol level in EMCV infected HEK-293T cells treated with curcumol and HPCD were detected using Amplex™ Red Cholesterol Assay Kit. The antiviral effects of curcumol and HPCD on EMCV were also quantitatively detected by real-time fluorescence quantitative PCR (q-PCR). The amount and morphology of ROs were observed by transmission electron microscopy (TEM).

**Results:**

The results demonstrated that curcumol significantly (*P* < 0.05) increased the expression of JAK1, IRF9, P-STAT2 and CH25H proteins, while that of STAT2, PI4KA and OSBP were remained unchanged. Compared with virus group (0.134 μg.μg^-1^ proteins), the total cholesterol level was significantly (*P* < 0.05) reduced by curcumol (0.108 μg.μg^-1^ proteins) and HPCD (0.089 μg.μg^-1^ proteins). Compared with virus group (88237 copies), curcumol (41802 copies) and HPCD (53 copies) significantly (*P* < 0.05) reduced EMCV load. Curcumol significantly reduced the production of ROs in EMCV-infected HEK-293T cells and activated CH25H through the JAK/STAT signaling pathway.

**Conclusion:**

Curcumol inhibited EMCV replication by affecting the cholesterol homeostasis and the production of ROs in HEK-293T cell.

**Supplementary Information:**

The online version contains supplementary material available at 10.1186/s12917-022-03531-x.

## Background

Encephalomyocarditis virus (EMCV) is a nonenveloped, single-stranded positive sense RNA virus in the *Picornaviridae* family. EMCV infection causes reproductive failure in sows, and acute myocarditis and sudden death in piglets [1–3]. Previous studies have shown that EMCV 3C protease can hydrolyze TRAF family member-associated NF-κB activator (TANK), interfere with the formation of TANK-TBK1-IKKε-IRF3 complex, and inhibit the production of type I interferon [4]. Our previous results showed that curcumol significantly inhibits the replication of EMCV, alleviates the hydrolysis of TANK protein by EMCV, and increases the expression of IFN-β in EMCV infected HEK-293T cells [5]. IFN-β has no direct antiviral activity, however it exerts an antiviral effect by activating the JAK/STAT signaling pathway and finally inducing the expression of interferon-stimulated genes (ISGs). It remains unknown which ISGs induced by curcumol is critical for inhibition of EMCV replication.

Cholesterol-25-hydroxylase (CH25H), an important ER-related enzyme that oxidizes cholesterol to 25-Hydroxycholesterol (25HC), is involved in cholesterol and lipid metabolism. CH25H is an interferon-stimulated gene and its primary product 25HC has antiviral activity against porcine reproductive and respiratory syndrome virus (PRRSV) [6], pseudorabies virus (PRV) [7] and porcine epidemic diarrhea virus (PEDV) [8]. Studies have shown that CH25H [9] and the cholesterol inhibitor HPCD [10] can significantly inhibit EMCV replication by reducing host cell cholesterol. It was reported that 200 approved drugs have been identified to be appropriate for repurposing against corona virus disease 2019 (COVID-19) using large-scale drug-induced gene expression profiles and 36% of the active compounds regulate those genes which are related to cholesterol homeostasis [11].

The picornaviruses genomic RNA replication occurs on specialized cellular remodeling membranes (single- or double-membrane vesicles) called ‘replication organelles’ (ROs). Virus-induced vesicles provide a structural platform to facilitate synergy between viral replication-related factors and also allow viruses to evade host defense systems [12, 13]. Its formation is related to viral non-structural proteins [14–18], but the specific mechanism of ROs formation is currently unclear. EMCV enters the cells and manipulates the lipid kinase phosphatidylinositol-4 kinase IIIα (PI4KA) to generate their ROs enriched with phosphatidylinositol 4-phosphate (PI4P)-4. PI4P recruits cellular oxidized cholesterol-binding protein (OSBP) to ROs. OSBP delivers cholesterol from ER to ROs with help of PI4P [19]. Studies have shown that ROs membrane cholesterol plays an important role in the efficiency of EMCV genome replication [10].

This study was designed to explore the effect of curcumol on CH25H and elucidate further explore whether the inhibition of ROs generation by curcumol is due to the interference of host cell cholesterol homeostasis.

## Results

### Curcumol activates CH25H via JAK/STAT signaling pathway

**Fig. 1:**
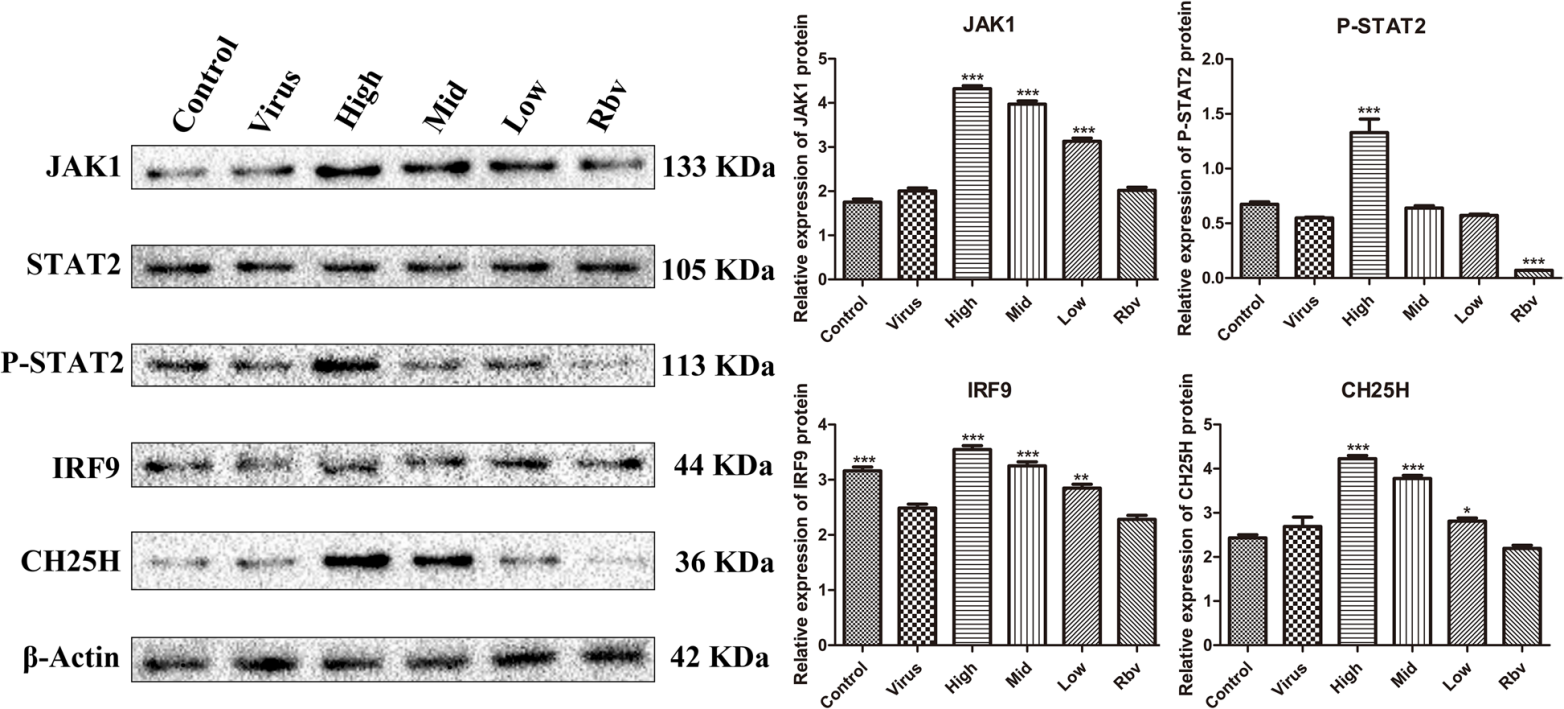
Curcumol activates CH25H via JAK/STAT signaling pathway. Three concentrations of curcumol (0.025, 0.0125 and 0.00625 mg/mL) and ribavirin (0.25 mg/mL) were selected to treat EMCV-infected HEK-293T cells for 24 h, and the expression of JAK1, STAT2, P-STAT2, IRF9 and CH25H protein were detected by Western blot. Original blots images were presented in Supplementary Figure 1

EMCV-infected HEK-293T cells were treated with curcumol and ribavirin for 24 h, respectively. The content of JAK1, STAT2, P-STAT2 and CH25H in the virus group was similar to that in control group, while IRF9 was significantly (*P* < 0.05) decreased (Fig. 1). The protein levels of IRF9, JAK1, P-STAT2 and CH25H in curcumol-treated group were significantly (*P* < 0.05) increased when compared to the virus group. Meanwhile, no significant change on the level of IRF9, JAK1, P-STAT2 and CH25H was observed in the ribavirin-treated group, compared to the virus group (Fig. 1). These results demonstrated that curcumol could activate JAK/STAT signaling pathway in the EMCV-infected HEK-293T cells.

### The Cholesterol affects EMCV replication

**Fig. 2:**
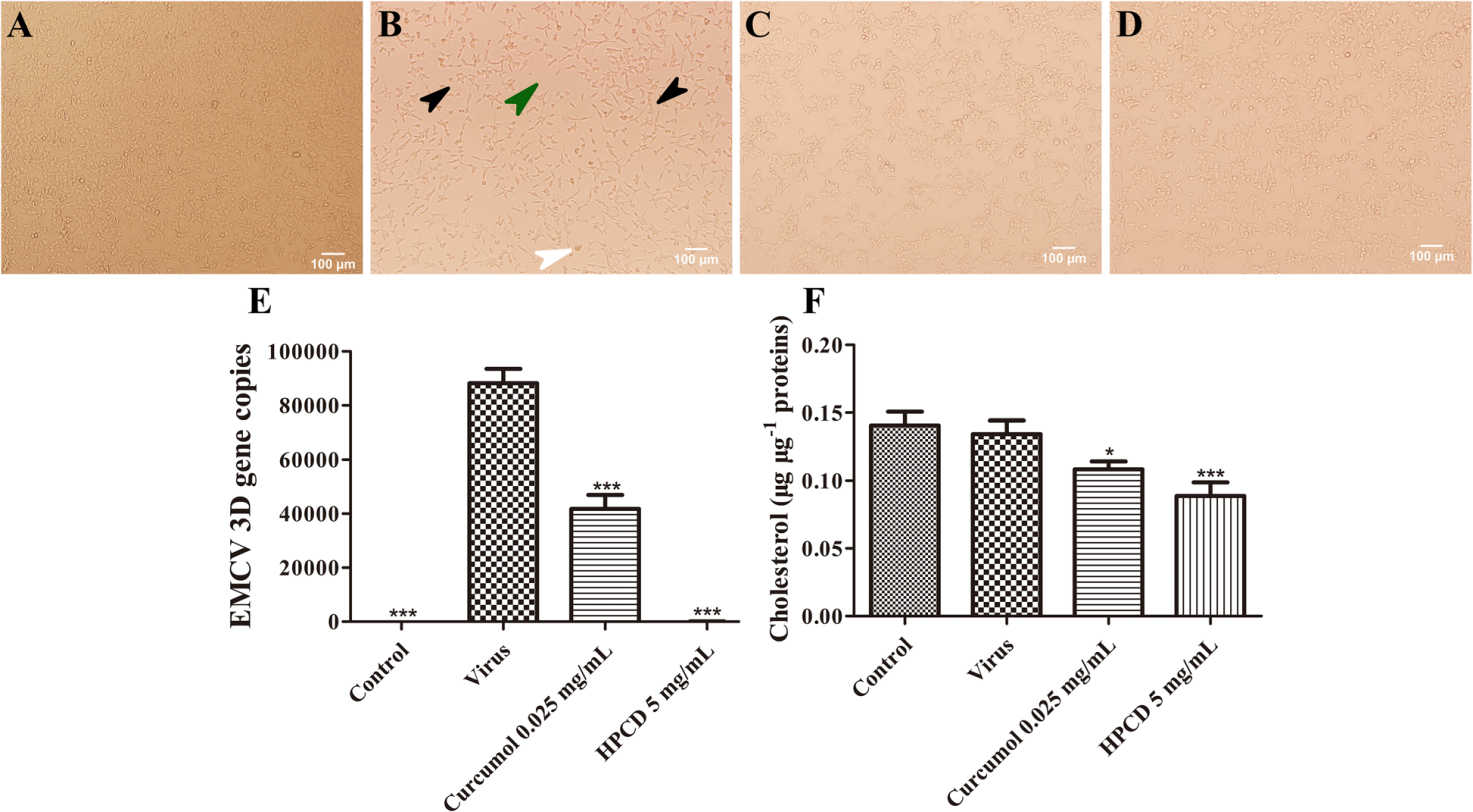
Curcumol reduces total cholesterol and inhibits EMCV replication. **A**-**D** The morphology of HEK-293 T cells post 24 h of treatment. A Control group, B Virus group, **C** Curcumol group, **D** HPCD group. **E** HEK-293T cells treated with curcumol and HPCD for 24 h, and the expression of the *3D* gene was detected by q-PCR. **F** Cholesterol depletion inhibits EMCV infection. HEK-293T cells treated with curcumol and HPCD for 24 h, and the total cholesterol was detected by Amplex™ Red Cholesterol Assay Kit

The viral load was detected by q-PCR after treating EMCV-infected HEK-293T cells with curcumol and HPCD for 24 h. Compared with control group (Fig. 2A), the cells monolayer shrink (black arrowheads), the cells become rounded (white arrowheads), detached (green arrowheads) and clumped together in virus group (Fig. 2B). The morphological changes observed in virus group were not present in the curcumol (Fig. 2C) and HPCD (Fig. 2D) treated groups. It indicated that both curcumol and HPCD significantly reduced the viral load (*P* < 0.05, Fig. 2E) and host cell cholesterol level (*P* < 0.05, Fig. 2F).

### Curcumol reduced the ROs generation

**Fig. 3:**
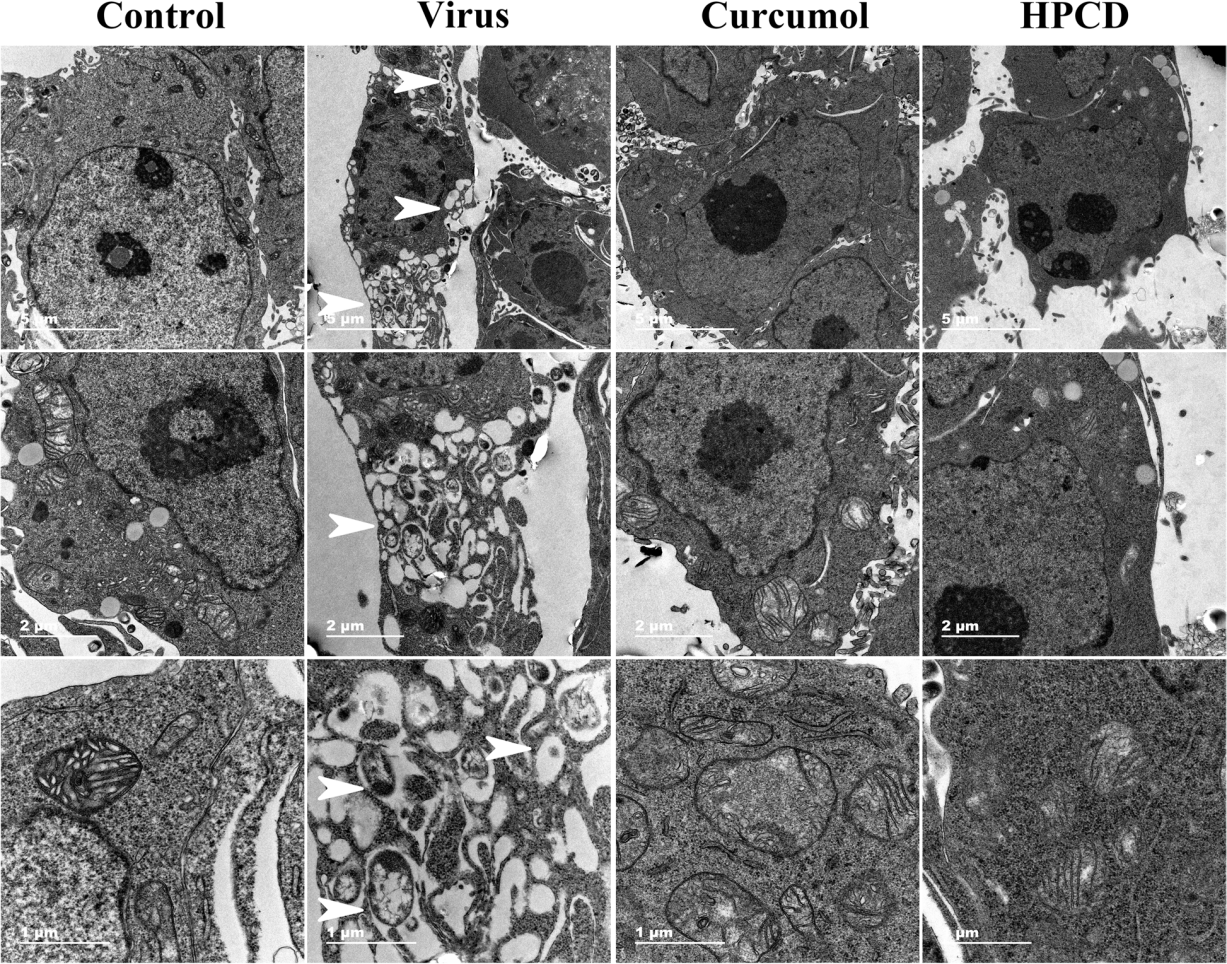
Curcumol inhibited the production of ROs. The production of ROs (white arrowheads) were detected by TEM in EMCV-infected HEK-293T cells treated with Curcumol (0.025 mg/mL) and HPCD (5 mg/mL). Scale Bars was 1 μm, 2 μm and 5 μm

The amount and morphology of ROs was detected by TEM after the EMCV-infected HEK-293T cells were treated with curcumol and HPCD for 24 h. The results showed that EMCV infection increased the ROs production significantly, while curcumol and HPCD treatments reduced the ROs level, indicating curcumol inhibited the ROs production (Fig. 3).

### Curcumol did not affect the level of PI4KA and OSBP

**Fig. 4:**
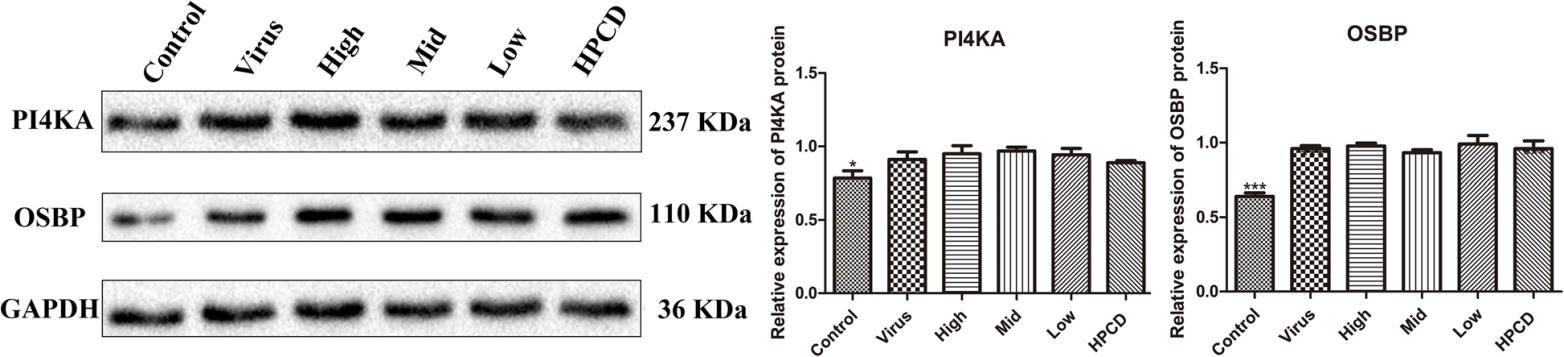
Curcumol did not affect the production of PI4KA and OSBP. Curcumol (0.025, 0.0125 and 0.00625 mg/mL) and HPCD (5 mg/mL) were selected to treat EMCV-infected HEK-293T cells for 24 h, and the expression of PI4KA and OSBP protein were detected by Western blot. Original blots images were presented in Supplementary Figure 2

PI4KA and OSBP protein were detected by Western blot after the EMCV-infected HEK-293T cells were treated with curcumol and HPCD for 24 h. EMCV induced the significant production of PI4KA and OSBP protein (Fig. 4). The level of PI4KA and OSBP protein in curcumol and HPCD treated groups was very similar to that in the virus group. The results showed that the inhibition of ROs production by curcumol was not related to PI4KA and OSBP.

## Discussion

Our previous results demonstrated that curcumol had anti-EMCV activity by alleviating the hydrolysis of TANK protein and increasing the expression of IFN-β in EMCV-infected HEK-293T cells [5]. IFN-β itself showed no direct antiviral activity, but do exert an antiviral effect by activating the JAK/STAT signaling pathway and inducing the expression of ISGs. As a member of ISGs, CH25H inhibits the EMCV replication in cell culture [9]. In this study, the antiviral mechanism of curcumol was explored in EMCV-infected HEK-293T cells treated with curcumol. Compared with the control group, the virus group showed no significant change in the expressions level of JAK1, STAT2, P-STAT2 and CH25H and the significant reduction in the IRF9 proteins. This is because the 3C protease expressed by EMCV inhibits the production of IFN-β [4]. However, the protein level of IRF9, JAK1, P-STAT2 and CH25H were significantly increased in EMCV-infected HEK-293T cells treated with curcumol for 24 h. These results demonstrated that curcumol significantly increased the expression of CH25H through activating the JAK/STAT signaling pathway.

Multiple mechanisms are involved in the antiviral effect of CH25H [6], including modulating inflammation, innate, and adaptive immunity and manipulation of cholesterol metabolism [20]. Studies have shown that 25HC inhibits the replication of enveloped viruses by blocking the fusion between the viral envelope and cell membrane [21]. EMCV has no envelope and 25HC inhibits EMCV infection by inhibiting the viral penetration [9]. Studies have shown that the cholesterol inhibitor HPCD significantly inhibit the replication of EMCV [10]. Our results showed that both curcumol and HPCD reduced the total cholesterol level and inhibited EMCV replication, possibly by activating CH25H and reducing total cholesterol in host cells.

**Fig. 5:**
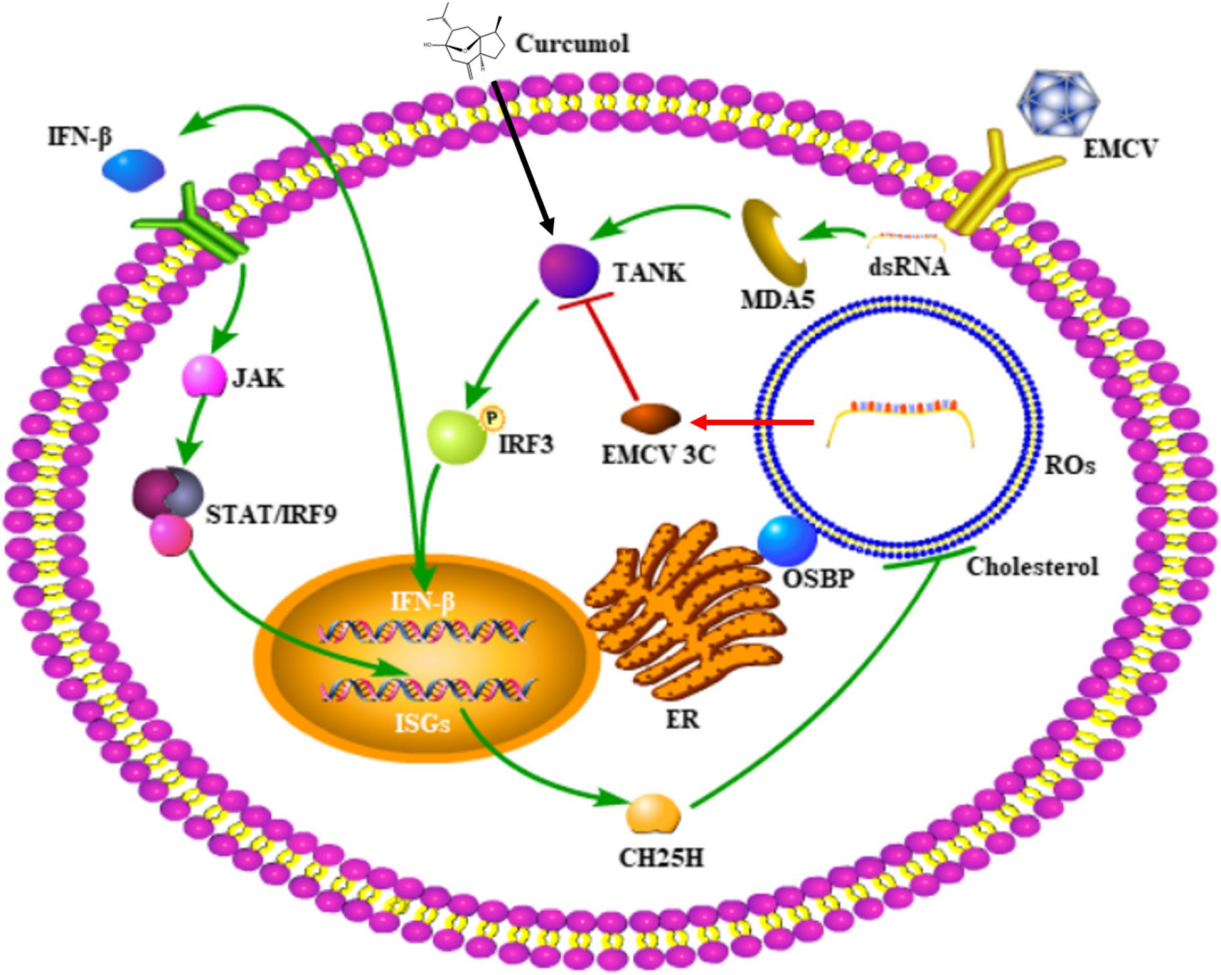
Schematic model of the mechanism of curcumol inhibiting EMCV replication. EMCV 3C protease can hydrolyze TANK and interfere with the formation of TANK-TBK1-IKKε-IRF3 complex, and inhibit the production of IFN-β. Curcumol can relieve the hydrolysis of TANK protein by EMCV 3C protease and promote the expression of IFN-β. IFN-β activated CH25H through the JAK/STAT signaling pathway. Curcumol inhibits EMCV replication is to reduce host cell cholesterol by activating CH25H and inhibiting the formation of ROs

ROs are associated with virus-induced membrane structures derived from different cellular compartments and crucial for the replication of all known picornavirus genomes. ROs of picornaviruses are associated with membranes that are derived from the ER [22]. Poliovirus-induced vesicles where viral RNA replication occurs were formed in the ER visualized by electron microscopic immunocytochemistry and autoradiography and then moved to the center of the infected cell to form a continuously growing vesiculated area for virus maturation [23]. PI4KB was recruited by viral 3A protein to enter ROs while PI4KA was recruited in EMCV infection [24–27]. The accumulation of PI4KB at ROs leads the local increase of the PI4P concentration, which is the synthesized product of PI4KB. This PI4P-rich environment further recruits other important viral and host factors to the replication site, such as the EMCV RNA polymerase 3D protein, which can specifically bind PI4P *in vitro*. PI4P recruits OSBP to ROs, and OSBP mediates the exchange of PI4P and ER cholesterol from ROs [19]. Studies have shown that membrane cholesterol on ROs can efficiently increase EMCV genome replication [10]. Our results shown curcumol caused the reduction of ROs and it was not related to PI4KA and OSBP. Therefore, it was concluded that curcumol inhibits EMCV replication by activating CH25H and interfering the ROs membrane cholesterol homeostasis (Fig. 5). Furthermore, this study showed that curcumol reduces the total cholesterol level of host cells. We will examine the effect of curcumol on ROs membrane cholesterol in our future studies.

## Methods

### Cell lines, viruses, plasmid, compounds and antibodies

HEK-293T (CL-0005) cells were kindly provided by Procell Life Science & Technology Co., Ltd. Cells were cultured in Dulbecco's modified eagle's medium (DMEM, Hyclone, USA) containing 10% fetal bovine serum (FBS, BI, Israel) (10% DMEM) and maintained in DMEM containing 2% FBS (2% DMEM).

The EMCV NJ08 strain (GenBank: HM641897) was gifted by Professor Jiang Ping of Nanjing Agricultural University. Virus was replicated and harvested using BHK-21 cells infected with EMCV. The titer of 10^8.5^ TCID_50_/mL was determined by MTT assay according to the method of Reed-Muench [28].

The recombinant plasmid containing the EMCV *3D* gene was generated in the laboratory [29]. Curcumol and ribavirin were purchased from China Food and Drug Control Institute, with 99.9% and 100% purity, respectively. HPCD was purchased from Sigma-Aldrich, with 100% purity. The dissolution of curcumol in DMEM requires 1% DMSO as a co-solvent, while ribavirin and HPCD are directly dissolved in DMEM.

STAT2 Rabbit Polyclonal antibody, JAK1 Ribbit Monoclonal antibody, IRF9 Rabbit Polyclonal antibody, GAPDH Mouse Monoclonal antibody, Beta Actin Recombinant antibody, Goat anti-Mouse and Goat anti-Rabbit secondary antibodies were purchased from Proteintech (USA). P-STAT2 and CH25H Rabbit Monoclonal antibody were purchased from Abcam (USA). PI4KA and OSBP Rabbit Polyclonal antibody were purchased from ABclonal (USA).

### Western blot analysis

The different protein levels in the HEK-293T cells infected with EMCV were detected by WB after the treatment with curcumol. Briefly, HEK-293T cells were seeded in 6-well plate and incubated with 100 TCID_50_/mL EMCV (2 mL/well) for 1.5 h. The supernatant was discarded and three 2 folds serial dilution of curcumol at starting dilution of 25 μg/mL were added and incubated for 24 h. The control group, virus group ribavirin positive control group (0.25 mg/mL) and HPCD (5 mg/mL) positive control group were applied.

The cells were lysed with RIPA buffer containing 1 mM protease inhibitor, 1 mM phosphatase inhibitor and cells were collected using a cell scraper. Total cell protein was extracted and protein concentration was determined using the BCA protein assay kit (Beyotime Biotechnology, Jiangsu, China). Equal amount of cell lysate was separated on a 10% SDS-polyacrylamide gel and transfer to a tailored polyvinylidene fluoride (PVDF) membrane according to the size of the protein. Then the membrane was blocked with Tris-buffered Tween 20 (TBST) with 5% non-fat dry milk at 25°C for 2 h. Next, the membrane was incubated with following primary antibodies overnight at 4°C: GAPDH Mouse Monoclonal antibody (1:50000), STAT2 and IRF9 Rabbit Polyclonal antibody (1:1000), PI4KA and OSBP Rabbit Polyclonal antibody (1:2000), JAK1and P-STAT2 Rabbit Monoclonal antibody (1:2000), CH25H Ribbit Monoclonal antibody (1:500). Then, the membrane was washed with TBST three times, and incubated with the membrane with goat anti-mouse and goat anti-rabbit secondary antibodies (1:20000) at 25°C for 2 h. Finally, the target protein was detected by an enhanced chemiluminescence system (Boster, China). Densitometric values of protein bands were quantified by the Image J.

### Real-time PCR

The HEK-293T cells cultured in 6-well plates were infected with 100 TCID_50_/mL EMCV, treated with same concentration of curcumol as above and incubated for 24 h. Total RNA was extracted from the cell samples, according to the Trizol protocol (Invitrogen, Carlsbad, CA, USA). The RNA concentration and purity were evaluated by Eppendorf BioPhotometer D30 (Eppendorf, USA). The complementary DNA was synthesized using PrimeScript® RT Master Mix kit with gDNA Eraser (TaKaRa, Dalian, China) according to the manufacturer’s protocol. Q-PCR was performed using a 7500 Real Time PCR System (ABI, USA). Absolute q-PCR was applied to determine EMCV *3D* gene level against the standard curve generated using serially diluted plasmid containing *3D* gene. The primer sequences used for EMCV *3D* gene were: F 5’-TTAGGGCGGGTTTG TAT-3’, R 5’-TTTGTTAGCGGGAGTTA-3’.

### Cholesterol quantification

The HEK-293T cells infected with 100 TCID_50_/mL EMCV (2 mL/well) and treated with 0.025 mg/mL curcumol and 5 mg/mL HPCD were incubated for 24 h. The cells were lysed with RIPA buffer containing 1 mM protease inhibitor, 1 mM phosphatase inhibitor. Cell protein concentration was determined by BCA assay (Beyotime Biotechnology, China). Cell lysates were normalized to equal amounts of protein for measurement of free and esterified cholesterol by the Amplex Red kit (Invitrogen, USA). The cholesterol standard was prepared following the manufacture’s instruction with cholesterol concentrations of 0 to 4 μg/mL (0 to 10 μM). 50 μL cell lysates diluted with reaction buffer was added to a microplate, followed by 50 μL working solution of 300 μM Amplex® Red reagent. The microplate was incubated at 37^o^C for 30 min and protected from light. The fluorescence was measured using a fluorescence microplate reader (Thermo Fisher Scientific, USA) with excitation at 560 nm and emission detection at 590 nm.

### Transmission electron microscope

The HEK-293T cells were cultured in 6-well plates, infected with 100 TCID_50_/mL EMCV (2 mL/well) and allowed to adsorb for 1.5 h. The supernatant was discarded, fresh medium with 0.025 mg/mL curcumol and 5 mg/mL HPCD were added for further incubation in a 5% CO_2_ 37°C cell incubator for 24 h. Meanwhile, control group, virus group were applied and incubated for 24 h. The cells were collected in a 1.5 mL centrifuge tube, and 2.5% glutaraldehyde solution was added (the centrifuge tube was filled with fixative solution so that the sample was completely immersed in the fixative solution), and the cells were fixed for 12 h. Pour off the fixative, rinse the sample three times with 0.1 M, pH 7.0 phosphate buffer for 15 min each time; fix the sample with 1% osmic acid solution for 2 h; carefully remove the osmic acid waste solution, rinse the sample three times with 0.1 M, pH 7.0 phosphate buffer for 15 min each time. The samples were dehydrated using increasing concentrations of ethanol (30%, 50%, 70%, 80%, 90% and 95%) for 15 min with each concentration and then treated with 100% ethanol for 20 min; finally transition to pure acetone treatment for 20 min. The samples were treated with the mixed V/V=1/1 solution of embedding medium and acetone for 1 h, the mixed V/V=3/1 solution for 3 h, and the pure embedding agent for overnight. The infiltrated samples were embedded and heated at 70°C overnight to obtain embedded samples. The samples were sectioned in an ultramicrotome (LEICA EM UC7 type) to obtain 70-90 nm sections, which were stained with lead citrate solution and uranyl acetate 50% ethanol saturated solution for 10 min each. After drying, it can be observed in a transmission electron microscope (Tecnai Spirit Bio-TWIN, FEI, USA).

### Statistical analysis

All data were presented as Mean ± SD. Data were analyzed using GraphPad Prism^TM^ software 5.0 (GraphPad Software, Inc. California, USA). Densitometric values of protein bands were quantified by the Image J. Data were analyzed using GraphPad Prism™ software 5.0 (GraphPad Software, Inc. California, USA). One-way analysis of variance (ANOVA) followed by a Dunnett’s post-test was used to determine the difference between the groups. All groups are compared with EMCV-infected group, * *P* < 0.05, ** *P* < 0.01, *** *P* < 0.001.

## Supplementary Information

**Additional file 1:** Supplementary Figure 1. Curcumol activates CH25H via JAK/STAT signaling pathway. Three concentrations of curcumol (0.025, 0.0125 and 0.00625 mg/mL) and ribavirin (0.25 mg/mL) were selected to treat EMCV-infected HEK-293T cells for 24 h, and the expression of JAK1, STAT2, P-STAT2, IRF9 and CH25H protein were detected by Western blot. Supplementary Figure 2. Curcumol did not affect the production of PI4KA and OSBP. Curcumol (0.025, 0.0125 and 0.00625 mg/mL) and HPCD (5 mg/mL) were selected to treat EMCV-infected HEK-293T cells for 24 h, and the expression of PI4KA and OSBP protein were detected by Western blot.

## Data Availability

The datasets used and analyzed during the current study are available from the corresponding author on reasonable request. Also, the datasets generated during the current study are available in the [Figshare] repository, [DOI:10.6084/m9.figshare.21076699].

## Abbreviations

CH25H: Cholesterol 25-hydroxylase
dsRNA: Double-stranded RNA
ER: Endoplasmic reticulum
EMCV: Encephalomyocarditis virus
HEK-293T: Human embryonic kidney 293T
IRF3: Interferon regulatory Factor 3
IRF9: Interferon regulatory factor 9
ISGF3: Interferon stimulated gene factor 3
ISGs: IFN-stimulated genes
IKK-ε: IKB kinases-ε
IFN-β: Interferon-β
JAK1: Janus kinase 1
MDA5: Melanoma differentiation- associated protein 5
MAVS: Mitochondrial antiviral signaling protein
TBK1: TANK-binding kinase 1
TANK: TRAF family member-associated NF-κB activator
OSBP: Oxysterol-binding protein
PI4KA: Phosphatidylinositol-4 kinase IIIα
ROs: Replication organelles
STAT1: Signal transducer and activator of transcription 1
