# Supplementary material for: Curcumol inhibits EMCV replication by activating CH25H and inhibiting the formation of ROs
Source: BMC Vet Res. 2022 Dec 26;18:453. doi: 10.1186/s12917-022-03531-x (PMC9791146; doi:10.1186/s12917-022-03531-x)
Supplement: Supplementary file 1 — Additional file 1: Supplementary Figure 1. Curcumol activates CH25H via JAK/STAT signaling pathway. Three concentrations of curcumol (0.025, 0.0125 and 0.00625 mg/mL) and ribavirin (0.25 mg/mL) were selected to treat EMCV-infected HEK-293T cells for 24 h, and the expression of JAK1, STAT2, P-STAT2, IRF9 and CH25H protein were detected by Western blot. Supplementary Figure 2. Curcumol did not affect the production of PI4KA and OSBP. Curcumol (0.025, 0.0125 and 0.00625 mg/mL) and HPCD (5 mg/mL) were selected to treat EMCV-infected HEK-293T cells for 24 h, and the expression of PI4KA and OSBP protein were detected by Western blot. [file 12917_2022_3531_MOESM1_ESM.pdf]

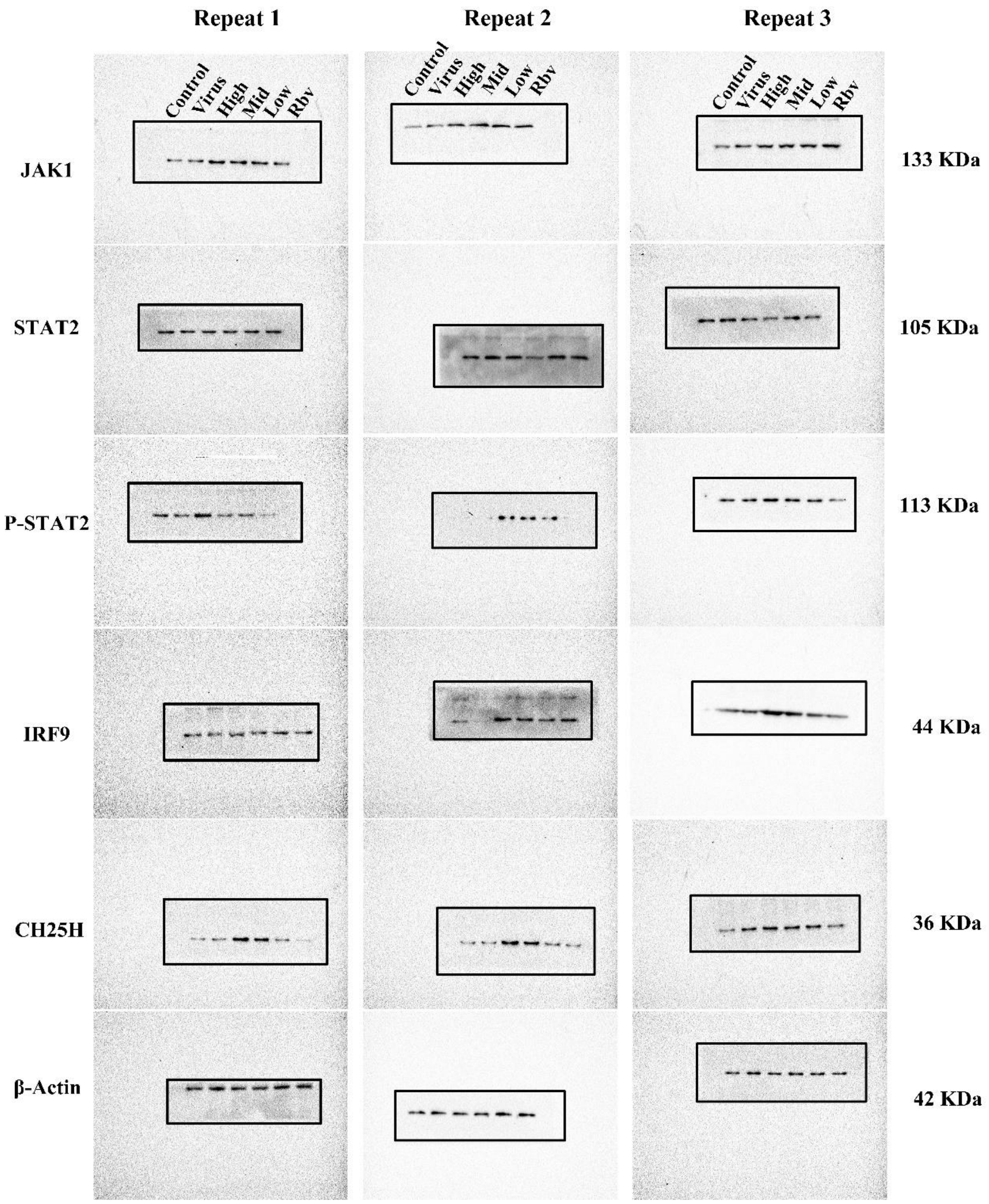

Supplementary Figure 1 Curcumol activates CH25H via JAK/STAT signaling pathway. Three concentrations of curcumol (0.025, 0.0125 and 0.00625 mg/mL) and ribavirin (0.25 mg/mL) were selected to treat EMCV-infected HEK-293T cells for 24 h, and the expression of JAK1, STAT2, P-STAT2, IRF9 and CH25H protein were detected by Western blot.

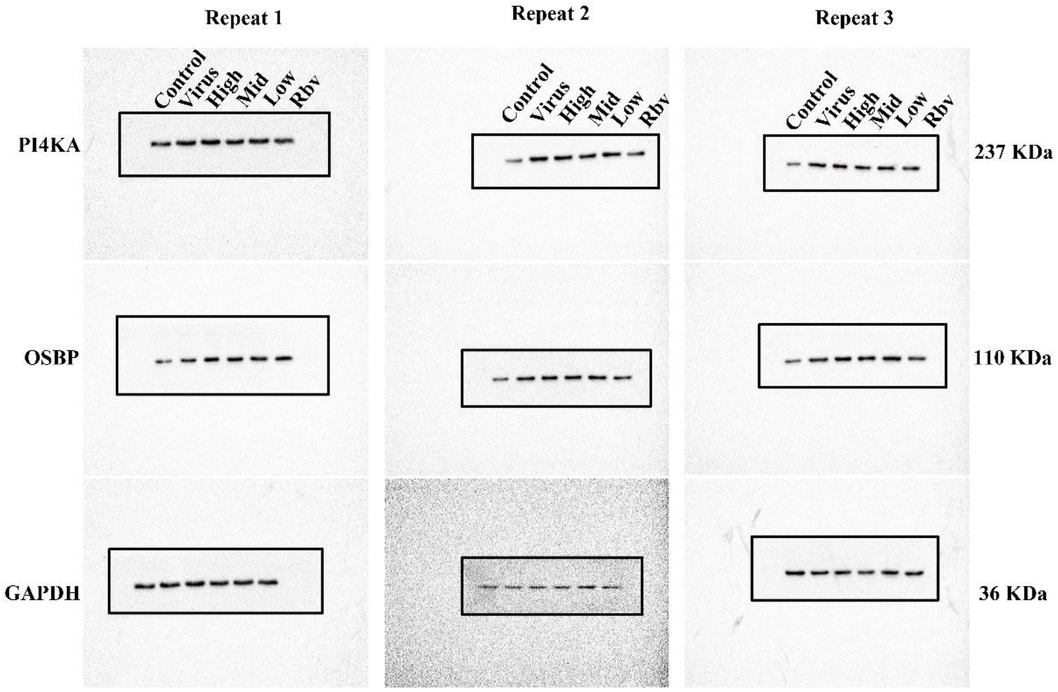

Supplementary Figure 2 Curcumol did not affect the production of PI4KA and OSBP. Curcumol (0.025, 0.0125 and 0.00625 mg/mL) and HPCD (5 mg/mL) were selected to treat EMCV-infected HEK-293T cells for 24 h, and the expression of PI4KA and OSBP protein were detected by Western blot.
